# Adult malaria mortality during 2019 at Bo Government Hospital, Sierra Leone

**DOI:** 10.12688/gatesopenres.14396.2

**Published:** 2023-08-14

**Authors:** Satta Sylvia T.K. Kpagoi, Ashley Aimone, Rashid Ansumana, Ibrahim Swaray, Hellen Gelband, John W. Eikelboom, Prabhat Jha, Isaac I. Bogoch

**Affiliations:** 1 Department of Medicine, Bo Government Hospital, Ministry of Health and Sanitation, Bo, Sierra Leone; 2 Centre for Global Health Research, Unity Health Toronto and Dalla Lana School of Public Health, Toronto, Ontario, Canada; 3 Health Sciences, Njala University, Bo, Sierra Leone; 4 McMaster University, Hamilton, Ontario, Canada; 5 Department of Medicine, University of Toronto, Toronto, Ontario, Canada

**Keywords:** Malaria, adults, mortality, Sierra Leone

## Abstract

It is uncertain whether malaria is an important cause of death among adults in endemic areas. We performed a chart review of adults admitted to Bo Government Hospital during 2019. Of 893 admissions, 149 (59% female, mean age 58.5 years) had a laboratory diagnosis of malaria and 22 (14.8%) died. Mortality was significantly higher among patients with severe malaria compared with those who had non-severe malaria (6/20 [30%] versus 16/129 [12.4%], *p*=0.031).  Our results suggest that malaria is a common cause of death in hospitalized Sierra Leonian adults.

## Introduction

It remains uncertain whether malaria is an important cause of death among adults living in endemic areas ^1^. One of the reasons for this uncertainty is that many countries with a high burden of malaria do not have a vital registration system. Malaria statistics for African countries published by the World Health Organization are based on the results of verbal autopsy in children under the age of five and provide little information on malaria mortality in adults ^2^.

We further explored the importance of malaria as a cause of death in adults in Sierra Leone by performing a chart review of all patients admitted to one of three adult medical wards at Bo Government Hospital during the 2019 calendar year. 

## Methods

We conducted a retrospective hospital-based chart review of adults (age ≥18) admitted to Bo Government Hospital from 1 ^st^ January to 31 ^st^ December 2019. Bo Government Hospital is major regional hospital in Southern Province, Sierra Leone and provides adult, maternal and pediatric care services to the city of Bo and surrounding districts. Three investigators (SK, AA, IB) extracted data from the medical charts of unselected consecutive adults admitted with a laboratory diagnosis of malaria during 2019 as part of the Countrywide Mortality Surveillance for Action (COMSA) Sierra Leone Project ^3^. The study included patients admitted over a 12-month period to provide a representative sample in view of the seasonal pattern of malaria. Eligible patients were identified based on review of laboratory results as documented in their medical charts and there were no exclusion criteria. Data extracted included age, sex (female/male), results of diagnostic testing for malaria (rapid diagnostic test, thick and thin film), information on the severity of malaria as defined by the World Health Organization criteria ^4^, and patient outcome (discharged alive or died in hospital).

Data were summarised as counts and mean and standard deviation for continuous variables, and number and percentage for categorical variables. The significance of any differences in proportions was evaluated with a chi square test and a 2-sided p value <0.05 was considered statistically significant. Analyses were performed using SAS version 9.4.

All investigators are listed as authors and had complete access to study data. 

Ethical permission was granted by the Office of the Sierra Leone Ethics and Scientific Review Committee (dated March 15, 2022). Individual patient consent was not required because the study involved a retrospective chart review.

## Results

Results are summarised in Table 1 ^5^. Of 893 adult admissions, 149 (59% female, mean age 58.5 years [SD 13.0]) had a laboratory diagnosis of malaria based on positive rapid diagnostic test or parasitemia on blood film. Of these, 20 (13.4%) were categorized as having severe malaria.

**Table 1.** Baseline characteristics and outcomes of patients admitted to Bo Government Hospital during 2019 with a diagnosis of malaria *.

Characteristic and outcome	Admissions with malaria (n=149)
Age, mean (SD)	58.5 (SD 13.0)
Age < 60	61 (52.6%)
Age ≥ 60	55 (47.4%)
Female, n (%)	86 (59.3%)
Hypertension	84 (66.1%)
Malaria severity, n (%)	
Non-severe	129 (86.6%)
Severe	20 (13.4%)
Deaths, n (%) †	22 (14.8%)
Severe	6 (30.0%)
Non-severe	16 (12.4%)

*Number of patients with missing data: age, n=33; sex, n=4 hypertension, n=22. †Comparison between severe and non-severe malaria: p=0.031 SD=standard deviation.

Of 149 patients with a malaria diagnosis, 22 (14.8%) died. There were no significant differences in age, sex or proportion of patients with hypertension among those whose chart review included criteria for severe malaria (including prostration, severe anaemia, jaundice, reduced level of consciousness, seizures, shock) compared with those who did not. Mortality was significantly higher among patients with severe malaria compared with those who had non-severe malaria (6/20 [30%] versus 16/129 [12.4%], *p*=0.031). 

## Discussion

The results of our chart review indicate that 1 in 7 adult patients admitted to Bo Government Hospital with laboratory confirmed malaria died. As expected, the mortality rate was highest in patients admitted with severe malaria, but most deaths still occurred in patients who did not have severe disease.

The large number of malaria admissions and high malaria mortality among adults observed during 2019 at Bo Government Hospital is consistent with emerging data that malaria is a significant contributor to adult mortality. The Sierra Leone Sample Registration System (SL-SRS) of births and deaths performed between September 1, 2019 and December 15, 2020 found that malaria accounted for 22% of deaths under the age of 70 years and was a leading cause of death in adults ^4^. Bittaye and colleagues reported 9.9% mortality in 131 adults (age 15–90) hospitalized with malaria during 2020–2022 in the Gambia, with all deaths occurring in patients with severe malaria ^6^. Boushab and colleagues reported 14.1% mortality in 99 adults (age >15 years) hospitalized with severe malaria during 2016–2019 in Mauritania ^7^. The Indian Million Death Study (MDS) suggested a higher adult mortality rate than expected, especially in persons aged over 45 years ^8^. Similar results were reported by the International Network for Demographic Evaluation of Populations and their Health (INDEPTH) Network ^9^.

Our data have some limitations. First, rapid diagnostic tests for malaria may be associated with both false positive and false negative results. It is likely that some adults had other underlying acute medical conditions leading to hospitalization but were categorized as having malaria due to an incidental finding of parasitemia on rapid testing or blood film examination. Second, classification of the severity of malaria was limited by lack of complete medical information, and it is possible that misclassification of the severity of disease explains the higher-than-expected mortality rate in those with non-severe malaria. Third, we did not have access to treatment information or the possible presence of drug resistance and are therefore unable to determine the reasons for the high adult malaria mortality in this study. It is also unclear whether concomitant infections, such as bacteremia, or other conditions may have contributed to malaria mortality seen in our study. Finally, our study was performed in a single regional hospital and the high mortality in hospitalized patients may not be generalizable to other hospitals in Sierra Leone.

In conclusion, our data demonstrate that the burden of adult malaria mortality in Bo Government Hospital, Sierra Leone is high. Further studies are needed to explore the reasons for the high mortality and more effective prevention and treatment strategies should be urgently implemented. 

## Data Availability

DRYAD: Health records from hospitalized adults with malaria during 2019 at Bo Government Hospital, Sierra Leone. https://doi.org/10.5061/dryad.5hqbzkh9s ^5^. This project contains the following underlying data: Data file 1: data extracted from retrospective medical chart review Data file 2: data dictionary README file: description of dataset Data are available under the terms of the Creative Commons Zero "No rights reserved" data waiver (CC0 1.0 Public domain dedication).
