# Supplementary material for: Adult malaria mortality during 2019 at Bo Government Hospital, Sierra Leone
Source: Gates Open Res. 2023 Aug 14;7:48. Originally published 2023 Mar 15. [Version 2] doi: 10.12688/gatesopenres.14396.2 (PMC10465729; doi:10.12688/gatesopenres.14396.2)
Supplement: Peer review history

# Peer review: Reviewer response for version 2

**DOI:** 10.21956/gatesopenres.16228.r34702

**Published:** 2023-08-29

**Authors:** Peter Okpeh Amede

**Affiliations:** 1 Nigeria Field Epidemiology and Laboratory Training Programme, Abuja, Nigeria

I have read the authors' responses to my comments and the revised manuscript. The authors have adequately responded to my comments and in areas authors differ, they provided reasons for it. 

 The manuscript is hereby **approved** for indexing.

Is the work clearly and accurately presented and does it cite the current literature?

Partly

If applicable, is the statistical analysis and its interpretation appropriate?

No

Are all the source data underlying the results available to ensure full reproducibility?

Yes

Is the study design appropriate and is the work technically sound?

Partly

Are the conclusions drawn adequately supported by the results?

Yes

Are sufficient details of methods and analysis provided to allow replication by others?

No

Reviewer Expertise:

NA

I confirm that I have read this submission and believe that I have an appropriate level of expertise to confirm that it is of an acceptable scientific standard.

# Peer review: Reviewer response for version 2

**DOI:** 10.21956/gatesopenres.16228.r34703

**Published:** 2023-08-25

**Authors:** Sheikh Omar Bittaye

**Affiliations:** 1 Department of Internal Medicine, Edward Francis Small Teaching Hospital, Banjul, The Gambia; 2 Internal medicine, University of the Gambia, Serrekunda, Banjul, The Gambia

I have no further comments.

 Thanks.

Is the work clearly and accurately presented and does it cite the current literature?

Partly

If applicable, is the statistical analysis and its interpretation appropriate?

Yes

Are all the source data underlying the results available to ensure full reproducibility?

Partly

Is the study design appropriate and is the work technically sound?

Yes

Are the conclusions drawn adequately supported by the results?

Yes

Are sufficient details of methods and analysis provided to allow replication by others?

Yes

Reviewer Expertise:

Infectious disease, hepatology, Gastroenterology

I confirm that I have read this submission and believe that I have an appropriate level of expertise to confirm that it is of an acceptable scientific standard.

# Peer review: Reviewer response for version 1

**DOI:** 10.21956/gatesopenres.15708.r33141

**Published:** 2023-05-03

**Authors:** Peter Okpeh Amede

**Affiliations:** 1 Nigeria Field Epidemiology and Laboratory Training Programme, Abuja, Nigeria

Congratulations to the authors for their manuscript. The study is about the burden of malaria among adults hospitalized in General Hospital Bo, Sierra Leone from 1 ^st^ January to 31 ^st^ December 2019. The study is part of the Countrywide Mortality Surveillance for Action (COMSA) Sierra Leone project.

**Listed below are points that require the authors’ attention to qualify the study.**

**Abstract: **Suggested reconstruction for consideration by the authors.

 It is uncertain whether malaria is an important cause of death among adults in endemic areas. We conducted a chart review of adults admitted to Bo General Hospital during 2019. Of the 893 adult admissions, 149 had laboratory diagnosis of malaria with mean (SD) age of 58.5 ±13.0 years, and most being female (59.0%). Among the laboratory confirmed malaria, 22 (14.8%) died. Mortality was significantly higher among patients with severe malaria compared with those who had non-severe malaria (6/20 [60%] versus (16/129 [12.4%], p=0.031). The finding suggests that malaria is a common cause of death among adults hospitalized in Bo General Hospital, Sierra Leone.

**Keywords: **Just one?

**Methods: **The authors should start the method section with the study design (We conducted a hospital-based retrospective chart review of malaria patients from 1 ^st^ January to 31 ^st^ December 2019. Three trained investigators ……..). A brief background of Bo General Hospital, Sierra Leone is required to bring readers up to speed on the study setting. The authors should define both the dependent and independent variables and how they were measured. The ethical permission should come just before data summary. The authors should explain clearly how one out of three medical wards was selected or there are three medical wards and all were included in the study? If so, the authors should please clarify. What level of significance was used for the study?

**Results: **The result section is quite scanty as in all the sections. The dataset has much information that were not analysed. For instance, is mortality higher among those with severe hypertension/elevated blood pressure? Or higher among the aged say 60 years and above? Among severe malaria cases how many are males/females? What about age distribution for severe malaria? Were there cases of malaria in pregnancy or they were excluded?

***Authors need to do more analysis from the available data, rather than clump everything as limitations. The data available is enough to provide solution/answers to most of what was put as limitations. Risk factor analysis could inform readers if age, sex, comorbidity are responsible for the severity or deaths. Also sensitivity analysis by excluding those with severe hypertension and sickle cell disease could have been helpful.***

**Discussion: **The discussion is not robust. The authors should discuss possible reasons for the high morbidity and mortality in the study, though it is a secondary data analysis, available data could provide reasons for the finding. And compare and contrast their findings with that of previous studies and discuss reasons for the similarity or otherwise between the studies.

 Limitations should be moved to the last paragraph of the discussion before the concluding paragraph.

**Conclusion from this study may not be generalizable; the authors need to limit the conclusion to the study setting, extending the findings of this study from a single hospital in one country to the entire Africa continent is superfluous.**

 My suggestion for the authors’ consideration. The study demonstrated that the burden of adult malaria mortality among malaria patients in Bo General Hospital, Sierra Leone was high. We therefore recommended that more effective prevention and treatment strategies be urgently implemented and there should be training and retraining of the healthcare workers by the health facility authority on proper documentation of medical records to improve data quality.

Is the work clearly and accurately presented and does it cite the current literature?

Partly

If applicable, is the statistical analysis and its interpretation appropriate?

No

Are all the source data underlying the results available to ensure full reproducibility?

Yes

Is the study design appropriate and is the work technically sound?

Partly

Are the conclusions drawn adequately supported by the results?

Yes

Are sufficient details of methods and analysis provided to allow replication by others?

No

Reviewer Expertise:

NA

I confirm that I have read this submission and believe that I have an appropriate level of expertise to confirm that it is of an acceptable scientific standard, however I have significant reservations, as outlined above.

# Response

**Published:** 2023-07-29

**Authors:** John Eikelboom

**Affiliations:** McMaster University, Canada

Congratulations to the authors for their manuscript. The study is about the burden of malaria among adults hospitalized in General Hospital Bo, Sierra Leone from 1 ^st^ January to 31 ^st^ December 2019. The study is part of the Countrywide Mortality Surveillance for Action (COMSA) Sierra Leone project.

Response: Thank you for these positive comments.

**Listed below are points that require the authors’ attention to qualify the study.**

**Abstract: **Suggested reconstruction for consideration by the authors.

 It is uncertain whether malaria is an important cause of death among adults in endemic areas. We conducted a chart review of adults admitted to Bo General Hospital during 2019. Of the 893 adult admissions, 149 had laboratory diagnosis of malaria with mean (SD) age of 58.5 ±13.0 years, and most being female (59.0%). Among the laboratory confirmed malaria, 22 (14.8%) died. Mortality was significantly higher among patients with severe malaria compared with those who had non-severe malaria (6/20 [60%] versus (16/129 [12.4%], p=0.031). The finding suggests that malaria is a common cause of death among adults hospitalized in Bo General Hospital, Sierra Leone.

Response: In the revised manuscript the abstract has been modified according to the suggestions of the Reviewer.

**Keywords: **Just one?

Response: In the revised manuscript additional key words have been provided.

**Methods: **The ****authors should start the method section with the study design (We conducted a hospital-based retrospective chart review of malaria patients from 1 ^st^ January to 31 ^st^ December 2019. Three trained investigators ……..). A brief background of Bo General Hospital, Sierra Leone is required to bring readers up to speed on the study setting. The authors should define both the dependent and independent variables and how they were measured. The ethical permission should come just before data summary. The authors should explain clearly how one out of three medical wards was selected or there are three medical wards and all were included in the study? If so, the authors should please clarify. What level of significance was used for the study?

Response: The methods section has been revised according to the suggestions of the reviewer. The statistical analyses are largely descriptive and test the significance of any differences between groups without any analyses that involve independent and dependent variables. We clarify that all adult general medical admissions were included.

**Results: **The result section is quite scanty as in all the sections. The dataset has much information that were not analysed. For instance, is mortality higher among those with severe hypertension/elevated blood pressure? Or higher among the aged say 60 years and above? Among severe malaria cases how many are males/females? What about age distribution for severe malaria? Were there cases of malaria in pregnancy or they were excluded?

***Authors need to do more analysis from the available data, rather than clump everything as limitations. The data available is enough to provide solution/answers to most of what was put as limitations. Risk factor analysis could inform readers if age, sex, comorbidity are responsible for the severity or deaths. Also sensitivity analysis by excluding those with severe hypertension and sickle cell disease could have been helpful.***

Response: In the revised manuscript additional analyses have been provided in relation to age, sex and hypertension. Unfortunately, data on comorbidities and on sickle cell disease are extremely limited which precludes further analysis.  

**Discussion: **The discussion is not robust. The authors should discuss possible reasons for the high morbidity and mortality in the study, though it is a secondary data analysis, available data could provide reasons for the finding. And compare and contrast their findings with that of previous studies and discuss reasons for the similarity or otherwise between the studies.

 Limitations should be moved to the last paragraph of the discussion before the concluding paragraph.

Response: The discussion has been elaborated along the lines suggested.

**Conclusion from this study may not be generalizable; the authors need to limit the conclusion to the study setting, extending the findings of this study from a single hospital in one country to the entire Africa continent is superfluous.**

Response: We agree and have modified the revised manuscript accordingly.  

 My suggestion for the authors’ consideration. The study demonstrated that the burden of adult malaria mortality among malaria patients in Bo General Hospital, Sierra Leone was high. We therefore recommended that more effective prevention and treatment strategies be urgently implemented and there should be training and retraining of the healthcare workers by the health facility authority on proper documentation of medical records to improve data quality.

Response: The conclusion of the revised manuscript has been modified along the lines suggested although we have stopped short of making recommendations regarding training / retraining of health care workers and regarding quality of health care records since we did not explicitly examine the possible contribution of these factors in the present study. We are currently in the process of further exploring these issues.

# Peer review: Reviewer response for version 1

**DOI:** 10.21956/gatesopenres.15708.r32984

**Published:** 2023-04-03

**Authors:** Sheikh Omar Bittaye

**Affiliations:** 1 Department of Internal Medicine, Edward Francis Small Teaching Hospital, Banjul, The Gambia; 2 Internal medicine, University of the Gambia, Serrekunda, Banjul, The Gambia

The authors provide some useful information about adult malaria mortality in Sierra Leone, specifically Bo Government hospital. However, the study has some inherent weaknesses. I have some comments and suggestions as to how the paper might be improved.

 1) It remains uncertain whether malaria is an important cause of death among adults living in endemic areas.

**Comment:** I doubt if this reflects the current situation. A lot of studies in endemic countries like Mauritania, Gambia, Uganda, Benin etc. have found malaria to have caused a significant burden in the adult age group.

 The statement could be true for Sierra Leone and thus could be stated as such and use the other studies already done to reference what they found.

 2) A chart review of all patients admitted to one of three adult medical wards at Bo Government Hospital.

**Comment:** The study used only one ward out of the three adult wards in Bo government hospital. It would have been better to look at all three wards to have a better representative of all adult admissions with malaria in Bo. 

 3. Execution in methodology

**Comment:** Change to Exclusion

 4. Adult definition

**Comment:** What's the definition for adult? 15 years and above?

 5.This number of severe malaria cases could have been underestimated due to limitations in availability of laboratory investigations in the diagnosis of severe malaria in Bo Hospital. The limitations in investigations should be highlighted and those diagnosed we should know the criteria used e.g how many were AKI, impaired conciousness etc.

Is the work clearly and accurately presented and does it cite the current literature?

Partly

If applicable, is the statistical analysis and its interpretation appropriate?

Yes

Are all the source data underlying the results available to ensure full reproducibility?

Partly

Is the study design appropriate and is the work technically sound?

Yes

Are the conclusions drawn adequately supported by the results?

Yes

Are sufficient details of methods and analysis provided to allow replication by others?

Yes

Reviewer Expertise:

Infectious disease, hepatology, Gastroenterology

I confirm that I have read this submission and believe that I have an appropriate level of expertise to confirm that it is of an acceptable scientific standard, however I have significant reservations, as outlined above.

## References

[1] : Clinical manifestations and outcomes of severe malaria in adult patients admitted to a tertiary hospital in the Gambia. *Malar J* .2022;21(1) : 10.1186/s12936-022-04294-4 270 10.1186/s12936-022-04294-4 36131306PMC9491657

# Response

**Published:** 2023-04-04

**Authors:** John Eikelboom

**Affiliations:** McMaster University, Canada

The authors provide some useful information about adult malaria mortality in Sierra Leone, specifically Bo Government hospital. However, the study has some inherent weaknesses. I have some comments and suggestions as to how the paper might be improved.

Author Response: Thank you for these thoughtful comments.

 1) It remains uncertain whether malaria is an important cause of death among adults living in endemic areas.

**Comment:** I doubt if this reflects the current situation. A lot of studies in endemic countries like Mauritania, Gambia, Uganda, Benin etc. have found malaria to have caused a significant burden in the adult age group.

 The statement could be true for Sierra Leone and thus could be stated as such and use the other studies already done to reference what they found.

Author Response: We agree with the reviewer that at a local level there is an appreciation of the burden of adult malaria mortality in many countries. However, this is less well appreciated by many others including the WHO, and accordingly we report that also in Sierra Leone adult malaria in higher than expected.  

 2) A chart review of all patients admitted to one of three adult medical wards at Bo Government Hospital.

**Comment:** The study used only one ward out of the three adult wards in Bo government hospital. It would have been better to look at all three wards to have a better representative of all adult admissions with malaria in Bo. 

Author Response: We apologize for the lack of clarity on this point. We did not restrict inclusion to patients admitted to one ward; rather we included patients admitted to any one of the three wards (i.e., all three wards were included). 

 3. Execution in methodology

**Comment:** Change to Exclusion

Author Response: We will correct this typographical error.

 4. Adult definition

**Comment:** What's the definition for adult? 15 years and above?

Author Response: Adults were defined as 18 years and above. 

 5.This number of severe malaria cases could have been underestimated due to limitations in availability of laboratory investigations in the diagnosis of severe malaria in Bo Hospital. The limitations in investigations should be highlighted and those diagnosed we should know the criteria used e.g. how many were AKI, impaired consciousness etc.

Response: We acknowledge that there are limitation sin testing protocols. The data on which these results are based are feely available on-line (see link in paper) and we would be pleased to provide additional details in the paper if required. 

- Is the work clearly and accurately presented and does it cite the current literature?
  Partly

Author response: We agree that not all current literature is cited reflecting our focus on the Sierra Leone results. We suggest that it would be timely to prepare a review article on adult malaria mortality that cites all of the recent literature and addresses potential strategies to address this (including adult immunization).      

- Is the study design appropriate and is the work technically sound?
  Yes
- Are sufficient details of methods and analysis provided to allow replication by others?
  Yes
- If applicable, is the statistical analysis and its interpretation appropriate?
  Yes
- Are all the source data underlying the results available to ensure full reproducibility?
  Partly

Author response: All available data are freely available on line

- Are the conclusions drawn adequately supported by the results?
  Yes

**References**

 1. Bittaye SO, Jagne A, Jaiteh LE, Nadjm B, et al.: Clinical manifestations and outcomes of severe malaria in adult patients admitted to a tertiary hospital in the Gambia. *Malar J*. 2022; **21** (1): 270 PubMed Abstract | Publisher Full Text
